# First report of therapeutic investigation and metabolic profiling of edible mushroom *Agaricus flocculosipes*

**DOI:** 10.1186/s13568-026-02028-2

**Published:** 2026-03-05

**Authors:** Divya Sreekala, Vipin Mohan Dan, Gama Mohan M. Geetha, Arya Chamaparambath, Anagha Bindu, Sajith Raghunandanan, Chittethu Kunjan Pradeep

**Affiliations:** 1 Division of Microbiology, KSCSTE- Jawaharlal Nehru Tropical Botanic Garden and Research Institute, Pacha-Palode, Thiruvananthapuram, Kerala 695562 India; 2 Department of Public Health and Genomics, Manipal School of Life Sciences, Manipal Academy of Higher Education, Manipal, Karnataka 576104 India

**Keywords:** Mushroom, *Agaricus flocculosipes*, *Streptococcus pyogenes*, Anticancer, Antibiofilm, Antimicrobial

## Abstract

The genus *Agaricus*, classified under the family *Agaricaceae* and order *Agaricales* of the class *Basidiomycota*, comprises nearly 500 species with a cosmopolitan distribution. This genus includes both edible and non-edible species, several of which hold significant nutritional and pharmacological value. Many of these edible mushrooms are well explored for their medicinal properties. *Agaricus flocculosipes*, is a recent addition in this genus introduced as an edible mushroom, but remains less explored for its therapeutic ability and commercial cultivation. This study for the first time explores medicinal properties of this mushroom. In the present study, *A. flocculosipes* exhibited selective inhibitory activity against Gram-positive pathogens, with the chloroform extract recording both Minimum Inhibitory Concentration (MIC) and Minimum Bactericidal Concentration (MBC) values at 50 µg/mL against *Streptococcus pyogenes*. Biofilm inhibition assays further demonstrated over 85% reduction in *S. pyogenes* biofilm formation at a concentration of 46 µg/mL. The chloroform extract also displayed potent anticancer activity, characterized by nuclear condensation, activation of caspase-9 and caspase-7, cleavage of Poly (ADP-ribose) polymerase (PARP), and upregulation of Bax, thereby inducing apoptosis in cancer cells. Metabolomic profiling of the chloroform fraction revealed a diverse array of bioactive compounds likely contributing to these observed biological effects. Collectively, these findings position *A. flocculosipes* as a promising medicinal mushroom with significant potential for future pharmacological and biotechnological research.

**Supplementary Information:**

The online version contains supplementary material available at 10.1186/s13568-026-02028-2.

## Introduction

Edible mushrooms are not only a rich source of essential nutrients but also exhibit functional properties, as their genomes encode diverse bioactive metabolites with potential therapeutic applications. Mushrooms are considered nutrient rich due to presence of high amount of protein, fiber, minerals, low fat content and vitamins like ascorbic acid, ergosterol, thiamine, riboflavin and cyanocobalamin (Hamza et al. 2024). In recent years, increasing awareness of their nutritional value, prophylactic and therapeutic benefits, as well as their use as adjuncts in disease management, has significantly enhanced their consumption. This growing demand has, in turn, driven the large-scale commercial cultivation of selected edible species.

The genus *Agaricus* comprises approximately 500 saprobic species with a global distribution, many of which are edible and highly valued for their nutritional richness, diverse medicinal properties, and suitability for large-scale commercial cultivation (Naritsada et al. 2016). Among these, *Agaricus bisporus*(J.E. Lange) Imbach and *Agaricus blazei* Murrill have been extensively investigated for their bioactive potential. *A. bisporus* is one of the most widely cultivated mushroom species globally and is valued for both its culinary and therapeutic applications. According to guidance from the Canadian Cancer Society, the consumption of *Agaricus bisporus* may enhance immune function and contribute to the prevention of various diseases (Ambhore et al 2024). The species contains numerous bioactive polysaccharides known to stimulate cellular immunity and exhibit antitumor effects (Usman et al. 2021). The antibacterial properties of *A. bisporus* can be correlated to presence of various compounds like Caffeic acid, Coumaric acid, Hydroxybenzoic acid, Azelaic acid, Gallic acid and Linolenic acid (Jankov et al. 2024). *A. blazei* Murill is another mushroom widely known and used for its range of therapeutic benefits. *A. blazei* Murill, first described from Florida, USA, has gained prominence as a natural therapeutic agent for managing several ailments, including cancer, hepatitis, arteriosclerosis, and diabetes. Its immunomodulatory effects are attributed to its ability to activate natural killer (NK) cells and promote leukocyte proliferation (Huang et al. 2022). Ergosterol and various polysaccharides isolated from *A. blazei* have shown efficient anti-tumor viability in animal models (Takaku et al 2001). The antimicrobial property of *A. blazei* has been more selective for gram-positive pathogens and recent studies have shown presence of antibacterial compounds like dibutyl phthalate, volemolide (17R)-17-methylincisterol and Linoleic acid (Yu et al. 2023; Cristiane et al. 2016). Cumulatively, existing literature indicates that the chemical composition of these edible *Agaricus* species can be closely associated with their diverse biological activities. *Agaricus flocculosipes*, a relatively recently described edible species within this genus, was first characterized from northern Thailand by Zhao and colleagues in 2012. To date, however, no comprehensive studies have investigated its therapeutic potential or elucidated its detailed chemical profile. Thus, as an unexplored edible species, the present study focuses on evaluating its medicinal properties, identifying its bioactive chemical constituents, and highlighting its significance as an edible medicinal mushroom.

Within section *Arvenses*, *A.flocculosipes* can be distinguished by its comparatively small basidiospores (< 6.5 µm in length), the presence of erect floccose squamules on the stipe surface, and its notably large sporocarps measuring 110–180 mm in diameter (Zhao et al. 2012). Thongklang et al. (2014) further demonstrated the species’ potential for commercial cultivation, reporting efficient mycelial colonization of compost and a relatively short fruiting period. However, they also observed low yield under existing cultivation conditions, suggesting that optimization of compost composition and modification of casing layer dimensions may enhance productivity. Despite over a decade since its initial discovery, the medicinal and functional properties of *A. flocculosipes* remain largely unexplored. The present study reports, for the first time, the therapeutic potential of this species by investigating its antimicrobial, antibiofilm, and anticancer activities. Metabolomic profiling of the bioactive fraction further elucidates the presence of key metabolites potentially responsible for these observed biological effects.

## Materials and methods

### Collection of fungal samples

The fungal samples of *A. flocculosipes* were collected from Western Ghats of India and transported in ice cold condition to laboratory. The integration of macro- and micromorphological features with nrITS-based phylogenetic analysis validated both the identity of the specimen and its systematic placement (Arya and Pradeep 2023). Once the macro- and micromorphological features were recorded, the basidiomata were dried overnight at 42 °C in a forced-air convection oven (30% humidity) and subsequently deposited in the Mycological Herbarium of Jawaharlal Nehru Tropical Botanic Garden and Research Institute [TBGT (M)] with specimen ID Arya TBGT(M) 17703, Arya TBGT(M) 17709 and Arya TBGT(M) 17721.

### Sample preparation and extraction

The fruiting bodies of mushroom specimens were dried in a dehydrator at 37–40 °C with constant air flow for five days. The dried samples were homogenized in a high-speed homogenizer until they turned into powder. A 10 g powdered sample was subjected to sequential Soxhlet extraction using 150 mL each of *n*-hexane, chloroform, ethanol, and methanol at their respective solvent boiling points. Each solvent extraction was carried out for 24 h, corresponding to eight extraction cycles per solvent. The extracts were concentrated at 45° C in rotatory evaporator and the samples were stored in -20˚C deep freezer until further use.

### Antimicrobial studies

#### Test organisms

The study employed three Gram-positive and one Gram-negative bacterial strains. The Gram-positive bacteria included *Streptococcus pyogenes* (ATCC 19615), *Staphylococcus aureus* (MTCC 443), and *Bacillus subtilis* (MTCC 441), while the Gram-negative bacterium was *Proteus vulgaris* (MTCC 426).

### Determination of antimicrobial activity of mushroom extract

The antimicrobial activity of mushroom extracts against selected pathogens was evaluated using the disc diffusion method (M Geetha et al. 2025). Bacterial cultures grown overnight were standardized to a concentration of 10⁸ CFU/mL. Sterile Mueller–Hinton Agar (MHA) plates (20 mL) were prepared, and the test organisms were evenly swabbed onto the surface. An aliquot of 10–15 µL of the crude mushroom extract dissolved in Dimethyl Sulfoxide (DMSO) was applied to each sterile disc placed on the inoculated plates, ensuring that each disc contained 500 µg of extract. Ciprofloxacin (5 µg) served as the positive control and DMSO alone in disc served as negative control. The plates were incubated overnight at 37 °C, and antibacterial activity was assessed by measuring the diameter of the growth-inhibition zones surrounding the discs.

### Determination of MIC (minimum inhibitory concentration) and MBC (minimum bactericidal concentration)

The Minimum Inhibitory Concentration (MIC) was determined using the microdilution method (Wiegand et al. 2008). In the present study, a predetermined non-toxic concentration of the solvent dimethyl sulfoxide (DMSO) was employed, with the final concentration maintained at or below 0.2% for all concentrations employed in study. Accordingly, the positive control contained 0.2% DMSO, a concentration non-toxic to the growth of the test pathogen. Microorganisms (10⁸ CFU/mL) were exposed to increasing concentrations (30 µg/mL to 60 µg/mL) of the mushroom extract in 24-well plates containing Brain Heart Infusion Broth (BHIB) as growth medium. Following overnight incubation at 37 °C, the lowest concentration that showed no visible microbial growth was recorded as the MIC. To determine the Minimum Bactericidal Concentration (MBC), a loopful from each tested concentration was streaked onto fresh Mueller–Hinton agar plates and incubated overnight at 37 °C. The lowest concentration that resulted in complete absence of growth was recorded as the MBC. The minimum bactericidal concentration (MBC) was further confirmed using the MTT assay at a working concentration of 0.5 mg/mL, following a 4 h incubation period, with absorbance measured at 570 nm, as described by Dan et al. (2024).

### Determination of MBIC (minimum biofilm inhibitory concentration)

Biofilm inhibition by *A*. *flocculosipes* chloroform extract was detected via crystal violet assay in 24 well plate. Chloroform extract in the range of 30 µg/mL to 60 µg/mL was employed to determine the MBIC value. Following treatment and a 24 h incubation period, the plates were rinsed thrice with 2 mL of distilled water and subsequently stained with 0.4% crystal violet. The bound dye was released with 80% glacial acetic acid. The percent biofilm inhibition was measured at 570 nm using a spectrophotometer. The assay was done in triplicate (N = 3). The following formula was used to determine the biofilm inhibition.

$$ \begin{aligned} & {\mathrm{Biofilm}}\;{\mathrm{inhibition}}\;\left( \% \right) \\ & = \left[ {\left( {{\mathrm{control}}\;{\mathrm{OD}}_{{{57}0\;{\mathrm{nm}}}} - {\mathrm{treated}}\;{\mathrm{OD}}_{{{57}0\;{\mathrm{nm}}}} } \right)/{\mathrm{control}}\;{\mathrm{OD}}_{{{57}0\;{\mathrm{nm}}}} } \right] \times {1}00 \\ \end{aligned} $$

### Light microscopic visualization of biofilm inhibition

For light microscopic visualization, *S. pyogenes* was allowed to grow in the presence or absence of mushroom extract on 1 × 1 cm glass slides in 24 well plate with Brain Heart Infusion Broth (BHIB). The glass slides were washed twice with 2 ml of distilled water and stained with 0.4% crystal violet and incubated for 10 min. The slides were washed again with 2 ml of distilled water and air dried. The dried glass slides were observed under light microscope.

### Anticancer studies

#### Anti-proliferative assay

The cervical cancer cell line SiHa was used to evaluate the anticancer efficacy of the chloroform extract. The antiproliferative activity of the chloroform extract was evaluated using the MTT assay ((4,5-dimethylthiazol-2-yl)-2,5-diphenyl tetrazolium bromide) in accordance with the standard protocol reported by Dan et al. (2024). Concentrations ranging from 1 to 40 µg/mL were used to determine the IC₅₀ value, and all assays were performed in biological as well as technical triplicates.

#### Nuclear condensation assay

Hoechst 33,342 staining was employed to assess nuclear condensation in treated cervical cancer cell line SiHa. Cells were seeded at a density of 10,000 cells per well in 24-well plates. Following 24 h incubation, the culture medium was aspirated, and cells were exposed to the determined IC₅₀ value of the chloroform extract. After 24 h of treatment, each well was incubated with Hoechst 33,342 (5 µg/mL) for 15 min at 37 °C in a CO₂ incubator. The cells were subsequently washed twice with PBS (Phosphate Buffered Saline) and visualized under a fluorescence microscope (Nikon, Japan) equipped with UV filter sets. Images were captured and analyzed using NIS-Elements (Version 0.410).

#### Western blot

Primary antibodies against PARP, Bax, Caspase-7, β-actin and Caspase-9 were obtained from cell signaling technology (USA). Protein was isolated from control and treated cell lines employing Radio-Immunoprecipitation Assay** (**RIPA) buffer (50 mM Tris–HCl, pH 8.0; 150 mM NaCl; 1% NP-40; 0.5% sodium deoxycholate; and 0.1% SDS) supplemented with phenylmethylsulfonyl fluoride (PMSF) as a protease inhibitor. Protein concentration was determined via Bradford assay. Equal amounts of protein (50 µg) from control and treated samples were denatured in SDS sample buffer by boiling for 6 min and subsequently subjected to SDS–PAGE. Proteins were resolved by SDS-PAGE (10–12%) and transferred onto a PVDF Polyvinylidene difluoride membrane. The membrane was blocked with 5% skim milk prepared in TBST (Tris-buffered saline with Tween-20) for 1 h at room temperature, followed by overnight incubation at 4 °C with the respective primary antibodies. After washing with TBST, the membrane was incubated with an HRP-conjugated anti-mouse secondary antibody for 1 h at room temperature. Immunoreactive protein bands were visualized using standard chemiluminescent detection protocols.

#### Metabolomic profiling

Vanquish UHPLC system equipped with a Waters RP Column (2.1 × 150 mm, 1.8 µm) was employed for chromatographic separations. The mobile phase consisted of 0.1% formic acid in water (A) and methanol (B). A gradient elution was initiated at 0.5% B with a flow rate of 350 µL min⁻^1^ over 15 min. The injection volume was 5 µL, and the autosampler temperature was maintained at 4 °C.

Mass spectrometric analysis was performed on an Eclipse Orbitrap with H-ESI, operated at a resolution of 60,000 and a scan range of m/z 70–1000. Data were processed in Compound Discoverer 3.3 using the *Untargeted Metabolomics* workflow. Solvent blanks were used to generate exclusion lists and remove background signals. compound identification relied on mzCloud and ChemSpider databases, with peak quality and elemental composition determined from isotopic fine structure of HRAM data.

### Statistical analysis

All experiments were performed in biological and technical duplicates or triplicates. Statistical significance was assessed using Student’s *t*-test with a threshold of *P* < 0.05. Nonlinear regression curve fitting was applied for control–treatment comparisons in the antiproliferation assay. Data analysis was conducted using GraphPad Prism version 9.0.0, and results are expressed as mean ± SEM.

## Results

### Antimicrobial activity and biofilm inhibition

**Fig. 1:**
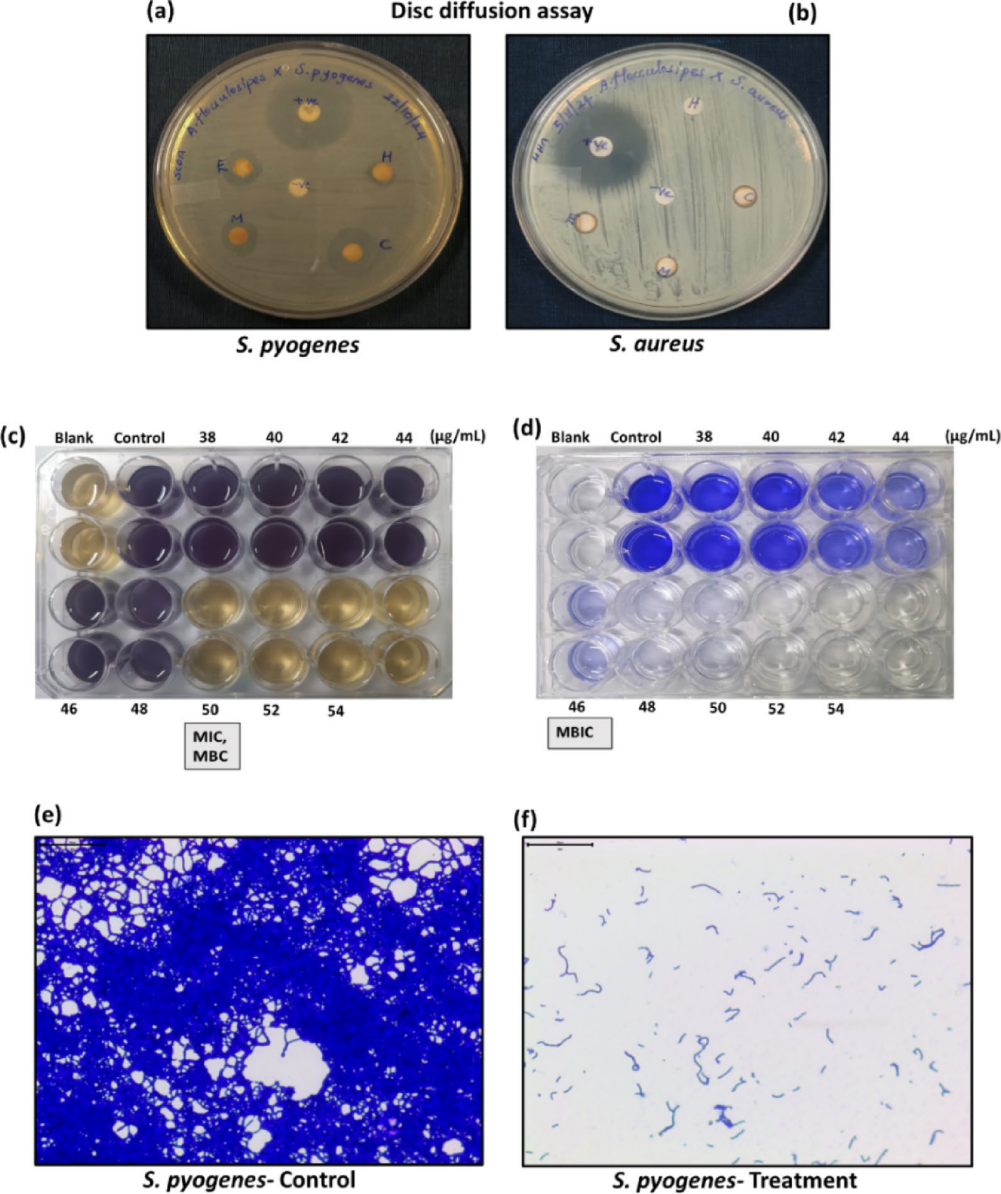
Antimicrobial and antibiofilm activity of *A. flocculosipes*: **a** Chloroform extract exhibited inhibitory activity against *S. pyogenes* and **b** *S. aureus*. c The minimum inhibitory concentration (MIC) and minimum bactericidal concentration (MBC) of chloroform extract were recorded at 50 µg/mL against *S. pyogenes*. MIC was determined by visual turbidity assessment, while MBC was confirmed using both MTT assay and agar streaking methods. **d** The minimum biofilm inhibitory concentration (MBIC) of chloroform extract was established at 46 µg/mL against *S. pyogenes* through crystal violet staining. **e–f** Microscopic analysis further demonstrated effective biofilm disruption and inhibition upon treatment with the chloroform extract against *S. pyogenes*. H-Hexane, M- Methanol, C- Chloroform, E- Ethanol

The mushroom extracts exhibited selective antimicrobial activity against Gram-positive pathogens, while no inhibitory effect was observed against the selected Gram-negative bacteria. The extract yield for each solvent is provided in supplementary information SI 1. Among the tested extracts, the chloroform fraction demonstrated the highest activity against *S. pyogenes*, with an inhibition zone of 14.70 ± 0.44 mm, whereas the hexane extract displayed the lowest activity with an inhibition zone of 6.90 ± 0.10 mm (Fig. 1a; Table 1). In contrast, the activity of the extracts against *S. aureus* was comparatively lower, with inhibition zones ranging between 6 and 7 mm (Fig. 1b; Table 1).

**Table 1:** Antimicrobial disc assay of four different extracts (500 µg of hexane, chloroform, ethanol and methanol) of *A. flocculosipes* against selected pathogens. Each experiment was performed in triplicates (N = 3)

Extracts	Zone of inhibition (mm)			
	*S. pyogenes*	*S. aureus*	*P. vulgaris*	*B. subtilis*
Hexane	6.90 ± 0.10	6 ± 0.12	–	–
Chloroform	14.70 ± 0.44	7.10 ± 0.21	–	–
Ethanol	12.40 ± 0.26	6.93 ± 0.10	–	–
Methanol	11.5. ± 0.29	7.23 ± 0.15	–	–
Ciprofloxacin	23.7 ± 0.44	22.5 ± 0.26	21.60 ± 0.31	24.17 ± 0.17

In the present study, *A. flocculosipes* demonstrated a stronger inhibitory effect against *S. pyogenes* compared to *S. aureus*. The chloroform extract showed the most potent antimicrobial activity among the tested fractions and was therefore selected for further investigations. The MIC of the chloroform extract against *S. pyogenes* was determined to be 50 µg/mL (Fig. 1c). MBC was also registered at 50 µg/mL against *S. pyogenes*. Additionally, the chloroform extract of *A. flocculosipes* effectively inhibited biofilm formation by *S. pyogenes*. Crystal violet assay registered 86.51 ± 0.94% inhibition of biofilm at 46 µg/mL (Fig. 1d). Light microscopic examination of biofilms formed in the presence and absence of the extract further confirmed the disruption and inhibition of biofilm development upon treatment (Fig. 1e and f).

### Anticancer activity

#### Antiproliferative activity and microscopic evaluation of apoptosis

**Fig. 2:**
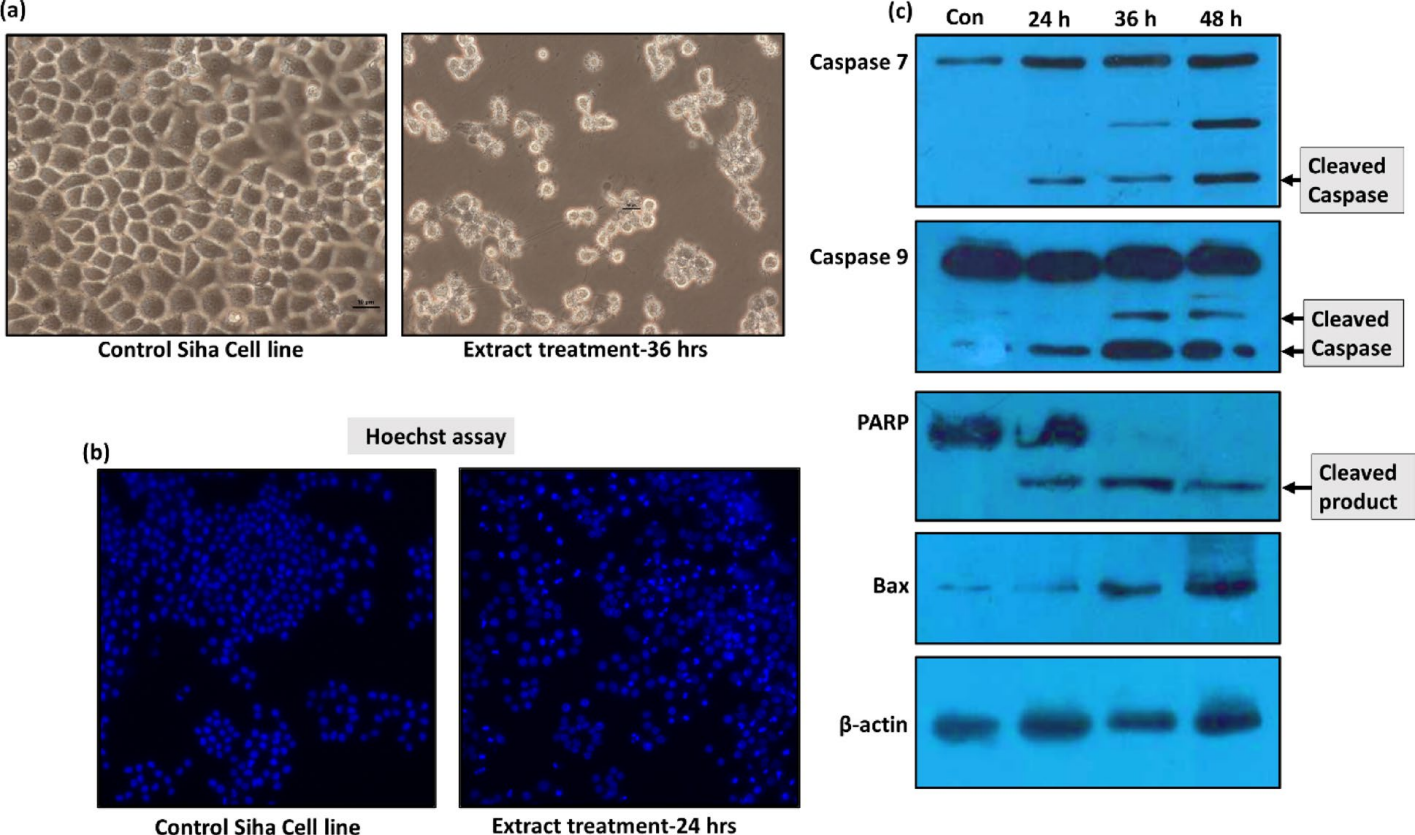
Evaluation of the anticancer potential of chloroform extract of *Agaricus flocculosipes* **a** Microscopic examination of treated cancer cells demonstrated characteristic morphological alterations probably associated to cell death, including cell rounding, membrane blebbing, and the formation of cytoplasmic spikes **b** Nuclear staining with Hoechst 33342 revealed pronounced chromatin condensation and nuclear fragmentation in treated SiHa cells **c** Immunoblot analysis of programmed cell death markers showed activation of caspases, poly (ADP-ribose) polymerase (PARP) cleavage, and increased accumulation of Bax protein. Full length blots of apoptotic markers and β-actin provided in Supplementary information SI 2

The MTT-based antiproliferative assay on the cervical cancer cell line SiHa revealed a concentration-dependent reduction in cell viability, with an IC₅₀ value of 15 ± 1.80 µg/mL for the chloroform extract. In the present study, treatment of cancer cell lines with the chloroform extract induced classical apoptotic features after 24 h and was clearly evident at 36 h (Fig. 2a). Morphological examination of treated cells revealed the formation of membrane blebs and spikes indicative of apoptosis, that was well evident at 36 h (Fig. 2a).

To further substantiate the occurrence of apoptosis, nuclear staining with Hoechst dye was performed. The chloroform extract-treated cells exhibited condensed chromatin localized within apoptotic bodies or membrane blebs (Fig. 2b), in contrast to the uniformly round nuclei with evenly distributed chromatin observed in untreated control cells. Collectively, these findings provide compelling evidence that the bioactive constituents present in the chloroform extract can potentially induce programmed cell death.

#### Analysis of apoptotic marker proteins

In the present study, Western blot analysis revealed the cleavage and inactivation of PARP-1 following treatment with the chloroform extract (Fig. 2c). 116 kDa full-length PARP-1was cleaved into an 89 kDa fragment (Fig. 2c). Furthermore, the activation status of caspase-9 and caspase-7 was examined upon chloroform extract treatment. Caspase-9 activation was confirmed by the presence of cleaved fragments, 37 kDa and 35 kDa, as detected by western blotting (Fig. 2c). Cleavage of full-length pro-caspase-7 (35 kDa) into its active 20 kDa form was also observed (Fig. 2c). Furthermore, Bax protein levels increased in a time-dependent manner upon extract treatment (Fig. 2c).

### Metabolomic profiling reveals therapeutic agents

**Fig. 3:**
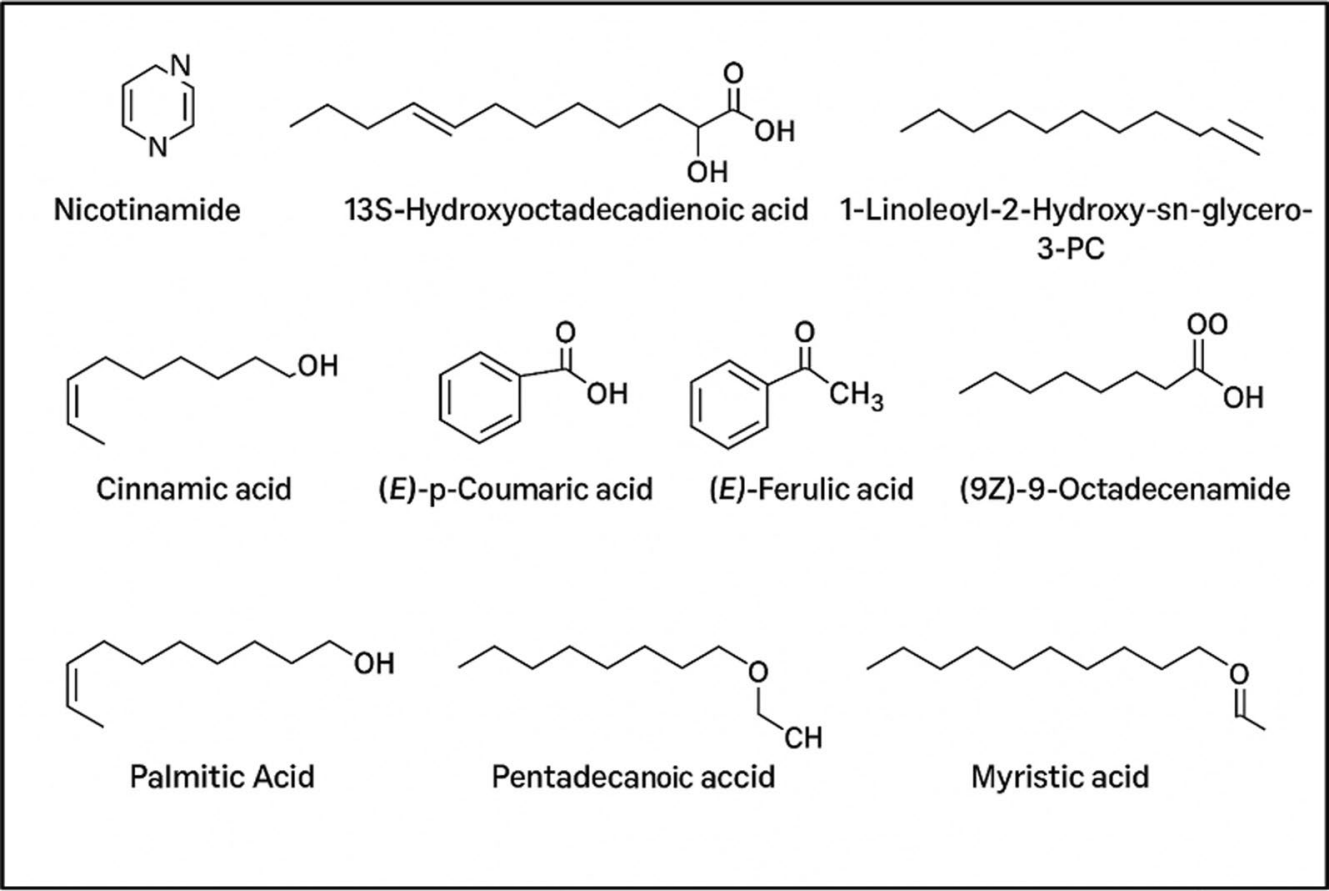
Chemical structures of bioactive compounds identified from the *A. flocculosipes* chloroform extract with therapeutic potential

Metabolic profiling of chloroform extract of *A. flocculosipes* revealed a diverse range of nutritional, functional, and bioactive constituents underscoring its culinary and medicinal significance. The analysis identified four major classes of compounds with therapeutic potential—fatty acids, fatty acid derivatives, phenolic acids, and vitamins/amides (Table 2a, Fig. 3). Among these, 13S-hydroxyoctadecadienoic acid and nicotinamide were the most abundant components (Table 2b, Fig. 3). Additionally, several nutritionally significant fatty acids and dietary bioactive such as oleic acid, coumaric acid, palmitic acid, ferulic acid, pentadecanoic acid, myristic acid, and cinnamic acid were detected, which may collectively contribute to the therapeutic effects observed in this study (Table 2b, Fig. 3).

**Table 2:** (a) Classification of compounds identified from the *A. flocculosipes* chloroform extract with therapeutic potential. (b) Relative abundance and physicochemical characteristics of therapeutically relevant compounds identified from the extract.

Class	Compounds
(a)	
Fatty acids	Palmitic, Pentadecanoic, Myristic, Oleic, 13S-hydroxyoctadecadienoic
Fatty acid derivatives	C1-linoleoyl-2-hydroxy-sn-glycero-3-phosphocholine, (9Z)-9-Octadecenamide
Phenolic acids	Cinnamic, p-Coumaric, Ferulic
Vitamins/amides	Nicotinamide

S.No	Name	Formula	Calculated M.W	Retention time	Area
(b)					
1	Nicotinamide	C_6_ H_6_ N_2_ O	122.0483	1.11	5.4E + 09
2	13S-hydroxyoctadecadienoic acid	C_18_ H_32_ O_3_	296.2357	10.254	2.9E + 09
3	1-Linoleoyl-2-Hydroxy-sn-glycero-3-PC	C_26_ H_50_ NO_7_P	519.3337	10.985	2E + 09
4	Oleic acid	C_18_ H_34_ O_2_	282.2564	10.997	6E + 08
5	Cinnamic acid	C_9_ H_8_ O_2_	148.0527	9.671	2E + 08
6	(9Z)-9-Octadecenamide	C_18_ H_35_ N O	281.2723	11.453	1E + 08
7	(E)-p-coumaric acid	C_9_ H_8_ O_3_	164.0476	6.394	1E + 07
8	Palmitic Acid	C_16_ H_32_ O_2_	256.2407	10.84	7E + 07
9	Pentadecanoic acid	C_15_ H_30_ O_2_	242.2251	10.567	1E + 06
10	(E)-Ferulic acid	C_10_ H_10_ O_4_	194.0584	8.731	2E + 06
11	Myristic acid	C_14_ H_28_ O_2_	228.2094	10.482	5E + 06

## Discussion

The nutritional and pharmacological potential of medicinal mushrooms belonging to the higher *Basidiomycetes* has garnered considerable scientific interest due to their diverse therapeutic applications. To date, more than seventy bioactive compounds have been isolated from the genus *Agaricus*, demonstrating a broad spectrum of biological activities, including anticancer, antioxidant, antimicrobial, and immunomodulatory effects (Zhang et al. 2024). The findings of the present study are consistent with earlier reports which revealed that organic extracts from closely related species, including *Agaricus essettei, Agaricus bitorquis*, and *Agaricus bisporus*, selectively inhibit Gram-positive bacteria (Öztürk et al. 2011). Similarly, extracts from *Agaricus devoniensis, Agaricus gennadii*, and *A. bisporus* exhibited preferential activity against Gram-positive pathogens, with *A. bisporus* showing stronger inhibition of *S. aureus* compared to the other two species (Soltanian et al. 2016). In our study the extract was more effective on *Streptococcus pyogenes* compared to *Staphylococcus aureus*, with the extracts of methanol, ethanol and chloroform registering inhibition zone above 10 mm. Soltanian et al. (2016) reported that the MIC₅₀ of wild *A. bisporus* extract was 39 µg/mL, while *A. devoniensis* and *A. gennadii* exhibited higher MIC₅₀ values of 1.25 mg/mL against *Staphylococcus aureus.* In contrast, the chloroform extract of *A. flocculosipes* exhibited both MIC and MBC values of 50 µg/mL, indicating greater antibacterial efficacy than that reported for *A. devoniensis* and *A. gennadii*. Notably, antimicrobial activity of *Agaricus* species against *S. pyogenes* has been scarcely documented.

The most abundant therapeutic metabolites in the chloroform extract, namely, Nicotinamide (NAM) ((Mattila et al. 2001), 13S-hydroxyoctadecadienoic acid (13S-HODE) (Batubara et al 2025) and 1-Linoleoyl-2-Hydroxy-sn-glycero-3-PC (Dong et al. 2022; Xie et al 2024) was earlier reported from various edible mushrooms. The other therapeutic metabolites identified were also reported from different edible mushrooms (Solomko et al. 1984; Saiqa et al. 2008; M Geetha et al. 2025; GünçErgönül et al. 2013; Radzki et al. 2024; Tan et al 2024; Abdalla et al. 2025). The presence of these bioactive compounds shared with other medicinally important edible mushrooms highlights the potential of *A. flocculosipes* and supports its further exploration as a functional food source.

Nicotinamide, cinnamic acid, *p*-coumaric acid, ferulic acid, 1-linoleoyl-2-hydroxy-sn-glycero-3-phosphocholine, and oleic acid have previously been reported to exhibit preferential antimicrobial activity against Gram-positive pathogens (Dilika et al. 2000; M Geetha et al. 2025; Guzman 2014; Miyazaki et al. 2017). The presence of these compounds in higher abundance may possibly contribute to the selective gram-positive antimicrobial nature of the studied chloroform extract. Nicotinamide (NAM) exhibits intrinsic antimicrobial activity and can also act synergistically to enhance the efficacy of other antimicrobial agents (AlSaleh et al. 2023). NAM may potentially contribute similar functional properties to the mushroom extract evaluated in the present study. Research studies have shown that Coumaric acid, Cinnamic acid and ferulic acid were more selective for *S pyogenes* than *S aureus* (Guzman 2014). These findings are consistent with our results, which demonstrate that the *A. flocculosipes* extract exhibited greater antibacterial efficacy against *S, pyogenes* than against *S. aureus*. The collective presence of these compounds in chloroform extract can potentially contribute to selective action on *S. pyogenes*. Earlier studies have proved that myristic acid and oleic acid can efficiently inhibit biofilm formation of *S. aureus* (Kim et al. 2022; Lee et al. 2022). In the present study, the chloroform extract effectively inhibited *S. pyogenes* biofilm formation, an effect that may be associated to the presence of the identified bioactive compounds; however, functional validation using purified compounds is required to confirm this association. It can also be stated that the antibiofilm activity of *Agaricus* species against *S. pyogenes* remains poorly explored, and the present study provides novel insights into their potential as effective antibiofilm agents.

The induction of programmed cell death observed in the present study can be potentially correlated with the presence of metabolites possessing established anticancer potential, as identified through metabolic profiling. Treatment with the mushroom extract resulted in the activation of effector caspases, specifically caspase-3 and caspase-7, along with the cleavage of PARP-1. During apoptosis, PARP-1 is a well-known substrate of effector caspases, primarily caspase-3 and caspase-7, with accumulating evidence suggesting a more prominent role for caspase-7 in PARP-1 cleavage (Germain et al. 1999). Several metabolites detected in the extract, including nicotinamide (NAM), 13S-HODE, and oleic acid, have been previously reported to induce apoptosis across a broad range of cancer types, such as breast cancer, melanoma, and colon cancer (Domínguez-Gómez et al. 2015; Scatozza et al. 2020; Yousef et al. 2022; Tavakoli et al. 2013; Jiang et al. 2017; Deng et al. 2023). In addition, compounds such as 1-linoleoyl-2-hydroxy-sn-glycero-3-phosphocholine (LPC), (9Z)-9-octadecenamide, *p*-coumaric acid, palmitic acid, cinnamic acid, and myristic acid have been shown to exert anticancer effects through inhibition of cancer cell proliferation and induction of apoptosis via caspase activation and BAX accumulation (Javid et al. 2023; Niero et al. 2013; Wang et al. 2023; Sharma et al. 2018; Tehami et al. 2023; Wisitpongpun et al. 2020; Zhang et al. 2023). These reports align closely with the findings of the present study, wherein treatment with the mushroom extract promoted caspase activation and BAX accumulation, suggesting the possible involvement of these compounds in the induction of mitochondrial-mediated apoptotic cell death.

## Conclusion:

The present study provides an initial insight into the therapeutic potential of the relatively underexplored species *A. flocculosipes*, demonstrating notable bioactivity and emphasizing its significance within the genus. The observed therapeutic effects may be attributed to the metabolites identified in the extract. Although earlier reports suggest favorable cultivation characteristics, systematic investigations into its pharmacological properties and agronomic optimization remain scarce. Collectively, these findings establish a foundation for future research focused on developing this mushroom as a functional food and elucidating additional bioactive constituents with therapeutic relevance.

## Supplementary Information

Below is the link to the electronic supplementary material.

Supplementary Material 1

## Data Availability

All data generated or analyzed during this study are included in this published article [and its supplementary information files].

## Abbreviations

PARP: Poly(ADP-ribose) polymerase
MIC: Minimum inhibitory concentration
MBC: Minimum bactericidal concentration
LPC: Lysophosphatidylcholine
MBIC: Minimum biofilm inhibitory concentration
MRSA: Methicillin-resistant *Staphylococcus aureus*
PVDF: Polyvinylidene difluoride
RIPA: Radio-immunoprecipitation assay
TBST: Tris-buffered saline with tween-20
