# Supplementary material for: First report of therapeutic investigation and metabolic profiling of edible mushroom Agaricus flocculosipes
Source: AMB Express. 2026 Mar 5;16:40. doi: 10.1186/s13568-026-02028-2 (PMC13069076; doi:10.1186/s13568-026-02028-2)
Supplement: Supplementary file 1 — Supplementary Material 1 [file 13568_2026_2028_MOESM1_ESM.docx]

**SUPPLEMENTARY INFORMATION (SI)**

**First report of therapeutic investigation and metabolic profiling of edible mushroom *Agaricus flocculosipes***

Divya Sreekala^a,#^, Vipin Mohan Dan^a,#,*^,[Gama Mohan M Geetha](https://pubmed.ncbi.nlm.nih.gov/?term=M+Geetha+GM&cauthor_id=40110618)^a^, Arya Chamaparambath^a^, Anagha Bindhu^a^, Sajith Raghunandanan^b.*^and [Chittethu Kunjan Pradeep](https://pubmed.ncbi.nlm.nih.gov/?term=Pradeep+CK&cauthor_id=40110618)^a,*^

^a^Division of Microbiology, KSCSTE- Jawaharlal Nehru Tropical Botanic Garden and Research Institute, Pacha-Palode, Thiruvananthapuram 695562, Kerala, India

^b^Department of public health and genomics, Manipal School of Life Sciences, Manipal Academy of Higher Education, Manipal, Karnataka - 576104, India

*Correspondence: Vipin Mohan Dan- [vipindan@gmail.com](mailto:vipindan@gmail.com), [vipindan@jntbgri.res.in](mailto:vipindan@jntbgri.res.in) ; Sajith Reghunandanan- [sajith.r@manipal.edu](mailto:sajith.r@manipal.edu) ;[Chittethu Kunjan Pradeep](mailto:Chittethu%20Kunjan%20Pradeep)-[pradeeptbgri@gmail.com](mailto:pradeeptbgri@gmail.com)

^#^Equal contribution

**Abstract**

The genus *Agaricus*, classified under the family *Agaricaceae* and order *Agaricales* of the class *Basidiomycota*, comprises nearly 500 species with a cosmopolitan distribution. This genus includes both edible and non-edible species, several of which hold significant nutritional and pharmacological value. Many of these edible mushrooms are well explored for their medicinal properties. *Agaricus flocculosipes*, is a recent addition in this genus introduced as an edible mushroom, but remains less explored for its therapeutic ability and commercial cultivation. This study for the first time explores medicinal properties of this mushroom. In the present study, *A. flocculosipes* exhibited selective inhibitory activity against Gram-positive pathogens, with the chloroform extract recording both Minimum Inhibitory Concentration (MIC) and Minimum Bactericidal Concentration (MBC) values at 50 µg/mL against *Streptococcus pyogenes*. Biofilm inhibition assays further demonstrated over 85% reduction in *S. pyogenes* biofilm formation at a concentration of 46 µg/mL. The chloroform extract also displayed potent anticancer activity, characterized by nuclear condensation, activation of caspase-9 and caspase-7, cleavage of Poly (ADP-ribose) polymerase (PARP), and upregulation of Bax, thereby inducing apoptosis in cancer cells. Metabolomic profiling of the chloroform fraction revealed a diverse array of bioactive compounds likely contributing to these observed biological effects. Collectively, these findings position *A. flocculosipes* as a promising medicinal mushroom with significant potential for future pharmacological and biotechnological research.

**Key words:** Mushroom**,** *Agaricus flocculosipes,* *Streptococcus pyogenes,* anticancer, antibiofilm, antimicrobial

| **S.No** | **Extract** | **Yield** |
| --- | --- | --- |
| 1 | Hexane | 62 mg |
| 2 | Chloroform | 72 mg |
| 3 | Ethanol | 68 mg |
| 4 | Methanol | 80 mg |

**SI 1:** Yield of extracts from the mushroom from 10 g *A. flocculosipes*. mg: milligram


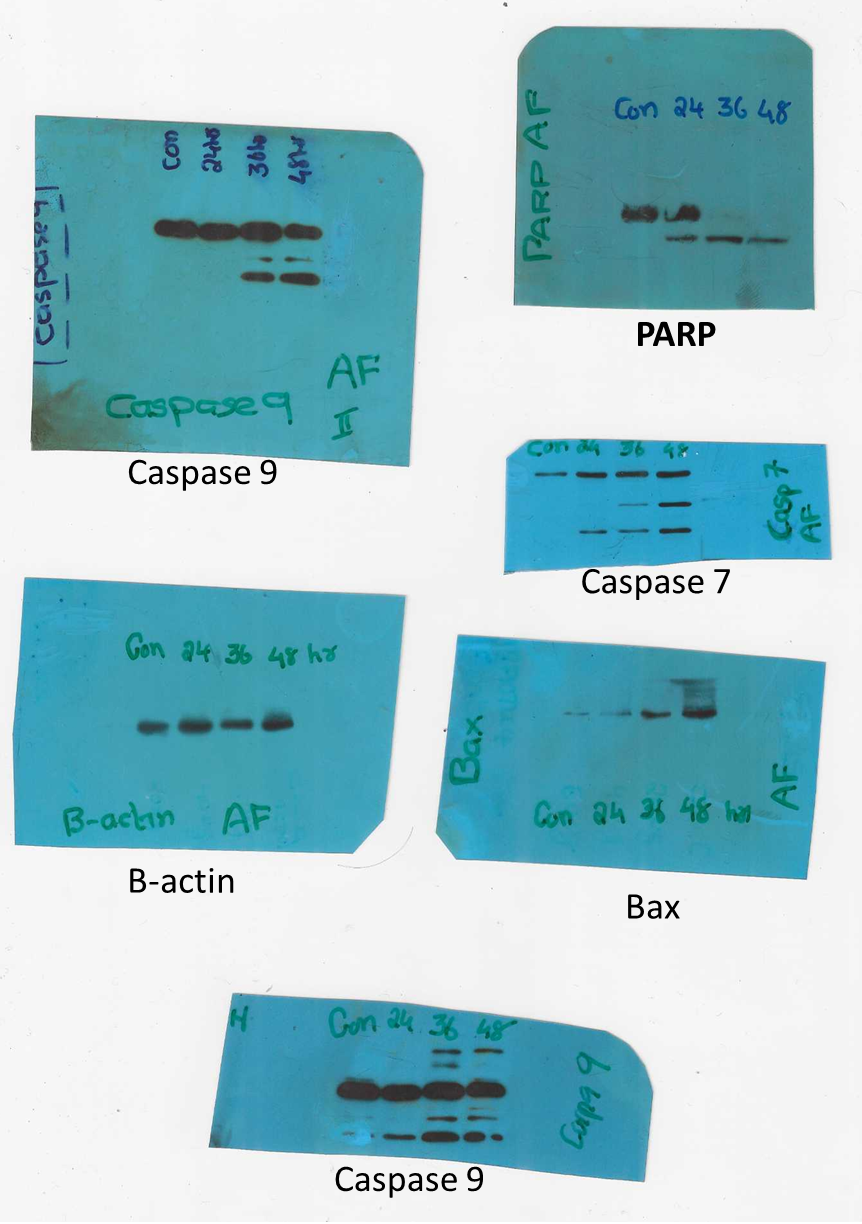


**SI 2**: Full length blots of various apoptotic markers and β-actin
